# Abdominal Cocoon With Intestinal Perforation: A Case Report

**DOI:** 10.3389/fsurg.2021.747151

**Published:** 2021-10-15

**Authors:** Qiang Hu, Jianfeng Shi, Yuanshui Sun

**Affiliations:** Department of General Surgery, Tongde Hospital of Zhejiang Province, Hangzhou, China

**Keywords:** abdominal cocoon, intestinal perforation, surgery, abdominal disease, case report

## Abstract

**Introduction:** Abdominal cocoon is a very rare abdominal disease. Abdominal cocoon mainly leads to intestinal obstruction, and abdominal cocoon with gastrointestinal perforation is rare.

**Case Presentation:** We report a 63-year-old man who was admitted to our hospital with “persistent lower abdominal pain for one day”. Abdominal CT examination revealed a small amount of free gas in the abdominal cavity, ascites, and gastrointestinal perforation. An emergency operation was performed. During the operation, the end of the right lower abdominal ileum was found to be conglutinated and twisted into a mass, a local intestinal dilatation, and obstruction, local intestinal wall was black and gangrene, and fecal effusion flowed out. The adhesions were carefully separated, and the necrotic small intestine was removed. The operation process went smoothly, and the patient recovered well after the operation.

**Conclusion:** The cases of intestinal perforation caused by the abdominal cocoon are very rare. In clinical work, when we encounter patients with gastrointestinal perforation, we need to carefully ask the history. When the patients had no digestive system diseases in the past, we need to consider the possibility of the abdominal cocoon with perforation.

## Introduction

Abdominal cocoon disease, also known as congenital small bowel incarceration and primary sclerosing peritonitis, is a rare abdominal disease with unclear causes and is difficult to diagnose. It has been reported that the misdiagnosis rate is as high as 95.4% (1). The abdominal cocoon disease is characterized by that part or all of the small intestine in the abdominal cavity is wrapped by white, dense, tough, and thick fibrous tissue, which is similar to silkworm cocoon in shape, and the greater omentum is absent. Due to the wrapping of fibrous tissue, the intestinal peristalsis is limited, so it is named as abdominal cocoon disease (2). The clinical manifestation of the abdominal cocoon is lack of specificity, and it is easy to be misdiagnosed before an operation, even endangering the life of the patient. We report a very rare case of the abdominal cocoon with small intestinal perforation and analyze its diagnosis and treatment.

## Case Presentation

**Figure 1:**
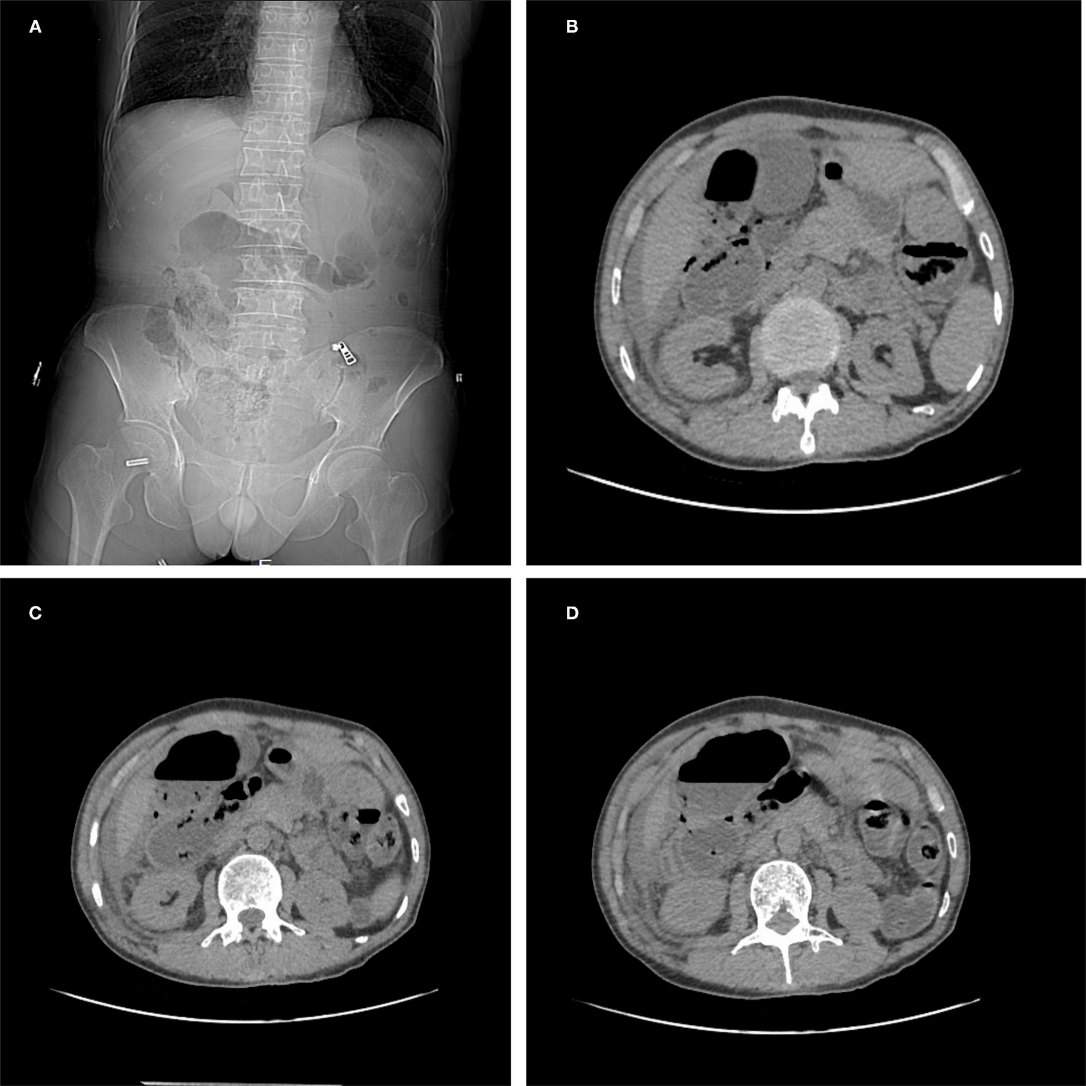
Abdominal CT examination **(A–D)**: a small amount of free gas in the abdominal cavity, ascites, gastrointestinal perforation.

A 63-year-old man was admitted to the hospital with “persistent lower abdominal pain for one day”. Physical examination: flat abdomen, plate-shaped abdomen, the tension of abdominal muscles, tenderness of the right lower abdomen, obvious rebound pain, negative mobile voiced sound, weak bowel sounds, one time/min. Imaging examination: abdominal CT examination: a small amount of free gas in the abdominal cavity, ascites, gastrointestinal perforation (Figure 1). Laboratory examination: white blood cell (WBC): 6.6^*^10E9/l, neutrophil (%): 90.8%, red blood cell (RBC): 3.49^*^10E12/l, hemoglobin: 70g/l, and C-reactive protein (CRP): 235.4 mg/l; direct bilirubin (DB) 7.8 μmol/l, total protein (TP): 35.9g/l, albumin (ALB): 22.1g/l, globulin13.8 g/l, sodium:135.2 mmol/l, amylase: 246U/l, procalcitonin (PCT) > 100.0 ng/ml. Previous history: the patient had a history of anemia, and the patients were treated with iron intermittently, and no obvious abnormality was found in the rest.

**Figure 2:**
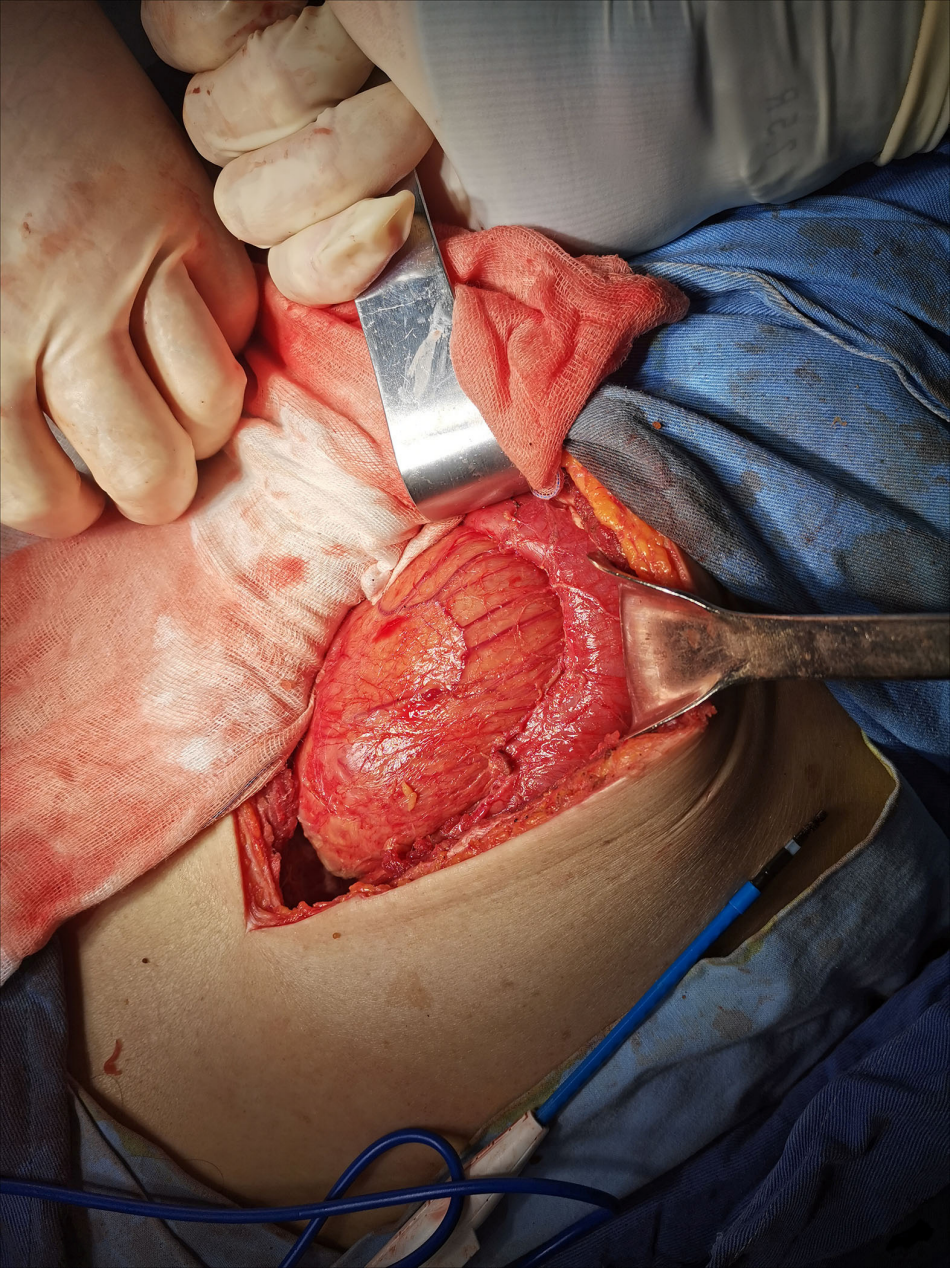
The end of the right lower abdominal ileum was found to be conglutinated and twisted into a mass.

**Figure 3:**
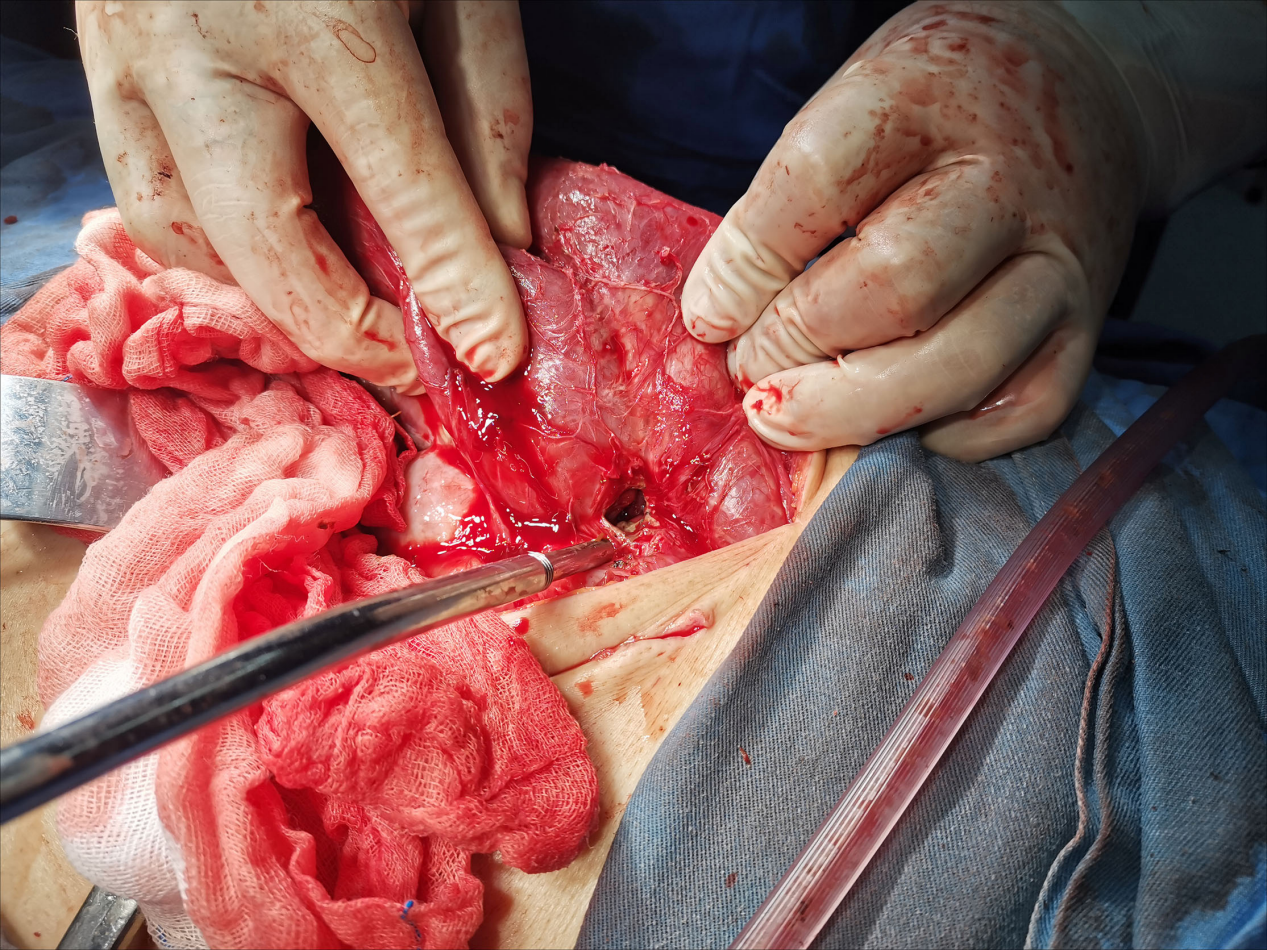
A gangrene rupture of the ileum was found about 50 cm from the ileocecal area.

**Figure 4:**
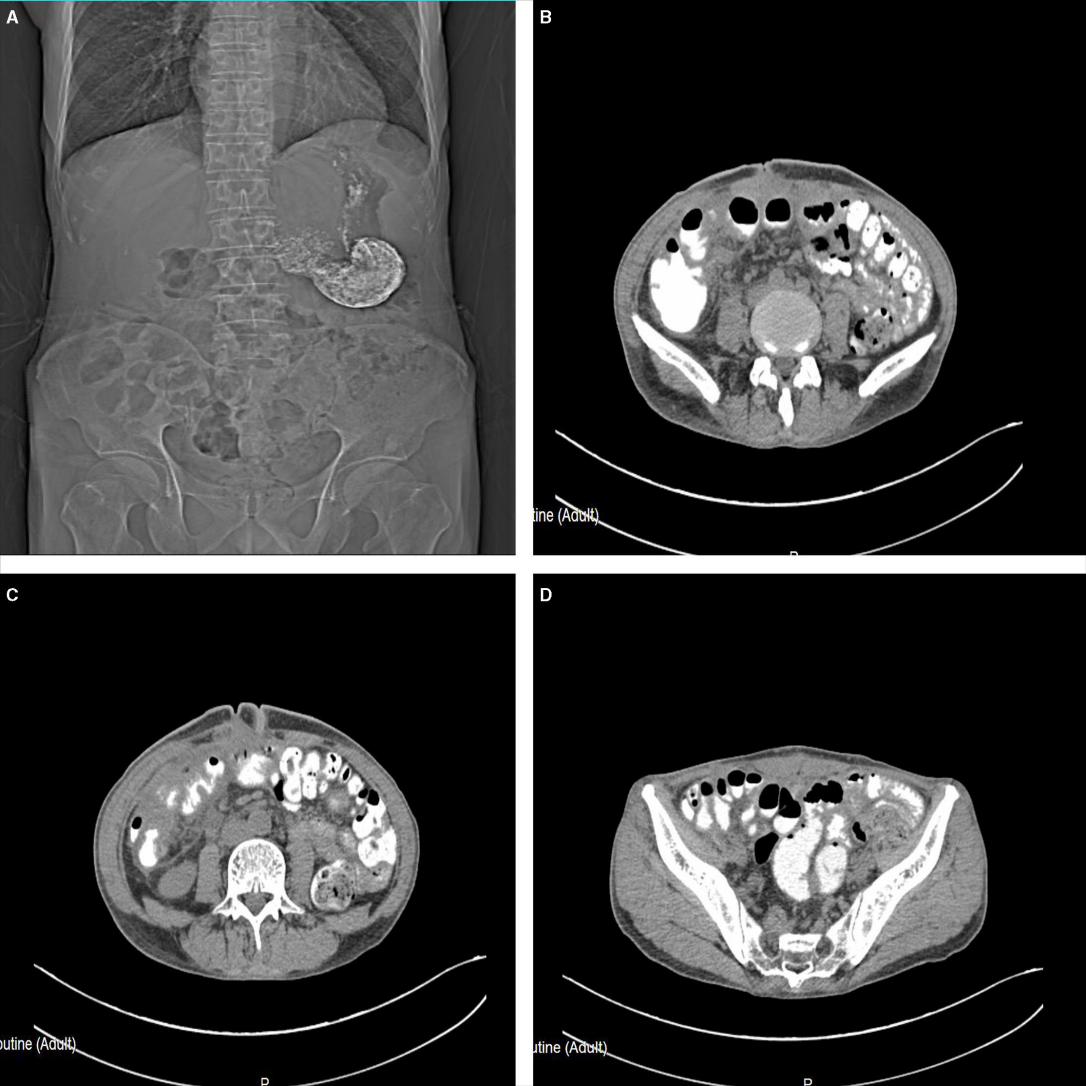
On the seventh day postoperation, the gastrointestinal angiography **(A–D)** showed that there was no leakage of the contrast agent in the abdominal cavity.

The patient was admitted to the hospital for emergency surgery, and the right side was dissected into the abdominal cavity through a rectus abdominis incision. During the operation, the end of the right lower abdominal ileum was found to be conglutinated and twisted into a mass (Figure 2). Local intestinal dilatation and obstruction were found, the local intestinal wall was black, and gangrene and fecal effusion were observed. The adhesions were separated with an electric knife and scissors, and a gangrene rupture of the ileum was found about 50 cm from the ileocecal area (Figure 3). The intestinal tube in the necrotic area was resected, about 20 cm long, and the distal and proximal small intestines were anastomosed side-to-side with a linear cutting closure device. Postoperative pathology: acute and chronic inflammatory cell infiltration, focal mucosal degeneration and necrosis, granulation tissue formation was found in the whole layer of the intestinal wall, immunohistochemistry: type IV collagen (+). Anti-infection and parenteral nutrition were given symptomatic treatment after operation. On the third day postoperation, the patient developed anal exhaust and was instructed to drink water properly. On the seventh day postoperation, the patient had anal defecation, and the patient was on a complete liquid diet, the gastrointestinal angiography showed that there was no leakage of contrast agent in the abdominal cavity (Figure 4). WBC:5.2^*^10E9/l, neutrophils (%): 71.6%, RBC: 3.82^*^10E12/l, hemoglobin: 91g/l, CRP: 15.6mg/l, TP: 57.5g/l, ALB: 34.8g/l, on the 10th day postoperation, the patients returned to their normal diet without abdominal pain and abdominal distension, and were discharged 12 days postoperation.

## Discussion

The abdominal cocoon disease is a rare peritoneum disease, which is characterized by a dense layer of gray-white membranous fibrous connective tissue wrapping some or all of the abdominal organs, similar to the cocoon of a silkworm. Because of the lack of specificity in the clinical manifestations, it is difficult to differentiate from the intestinal obstruction, perforation, or abdominal mass caused by the other reasons, so the preoperative diagnosis is more difficult, and the diagnosis is majorly based on the intraoperative findings (3, 4). The patient underwent an emergency operation because of the gastrointestinal perforation, and the abdominal cocoon was found during the operation.

Abdominal cocoon is more common in men, and the ratio of male to female is about 1.2-2:1 (5, 6). Abdominal cocoon is a rare peritoneal disease that was first reported and named by Foo et al. It is characterized by small intestinal obstruction caused by all or part of the small intestine covered by a dense fibrous membrane, also known as idiopathic sclerosing peritonitis, small intestinal incarceration, and small intestinal fibrous membrane entrapment (7, 8).

The causes of abdominal cocoon can be divided into primary and secondary abdominal cocoon disease (9). Primary abdominal cocoon disease is majorly caused by embryo body curling, abnormal mesoderm differentiation, and intestinal dorsal mesenteric dysplasia during the embryonic period. It is often accompanied by omentum absence, gastrocolic ligament absence, intestinal or colonic malrotation, visceral transposition, cryptorchidism, hernia, and other diseases (10). The causes of the secondary abdominal cocoon are as follows: (1) Abdominal microenvironment changes: an abdominal and pelvic infection caused by various reasons, history of abdominal surgery, long-term peritoneal dialysis, and malignant tumor, etc. Abdominal tuberculosis, autoimmune diseases, liver transplantation, and other immune factors can also induce abdominal cocoon (11). In recent years, some clinical workers also observed that abdominal injury and hepatitis C were the causes (12). There are also reported cases of the abdominal cocoon with abdominal free gas (13). (2) Drug effects: chemotherapy drugs, B receptor blockers, and mercury can induce peritonitis to form the abdominal cocoon, which is related to excessive collagen production and abdominal fibrosis (14–16). The patient had a history of anemia and no other disease was found, so it was considered as a primary abdominal cocoon.

The abdominal cocoon is characterized by abdominal pain, abdominal distension, vomiting, and abdominal mass. The duration of the disease varies. Preoperative diagnosis is difficult, and most of them are diagnosed based on intraoperative findings. Wei et al. reported that the main clinical manifestations of 24 cases of the abdominal cocoon were incomplete or complete intestinal obstruction (88%) and abdominal mass (54%) (17). Machado et al. reported that the main clinical manifestations of 118 cases of the abdominal cocoon included abdominal pain (72%), abdominal distension (44.9%), and abdominal mass (30.5%) (18). At present, there are few reports about abdominal cocoon with intestinal perforation.

Preoperative gastrointestinal radiography and high-resolution CT examination have a certain value in the diagnosis of the abdominal cocoon (19). However, gastroenterography may aggravate the abdominal symptoms in patients with acute intestinal obstruction or perforation, which should be cautious (20). CT diagnosis has high-technical requirements for doctors, this patient had an abdominal CT examination, but no abdominal cocoon was found, so the diagnosis of abdominal cocoon was mainly based on the intraoperative observation.

The principle of operation is to remove the capsule, release adhesion, and relieve the obstruction (21, 22). The operation methods include the following: (1) Intestinal resection: it is suitable for cases of intestinal ischemia and necrosis. In this case, intestinal necrosis occurred, so we removed part of the necrotic small intestine. (2) Intestinal arrangement surgery: it is suitable for the cases with severe adhesion and cannot be separated, so as to relieve the obstruction. It should not be widely separated in order to completely remove the capsule, so as to avoid causing too many other injuries, resulting in the postoperative intestinal fistula, necrosis, and other complications. (3) Appendectomy: it is suitable for the patients with obvious appendiceal fecal stone found in operation and the possibility of acute appendicitis in the later stage.

## Conclusions

The cases of intestinal perforation caused by the abdominal cocoon are very rare. In clinical work, when we encounter patients with gastrointestinal perforation, we need to carefully ask the history. When the patients do not suffer from any digestive system diseases in the past, we must consider the possibility of the abdominal cocoon with perforation.

## Data Availability Statement

The original contributions presented in the study are included in the article/supplementary material, further inquiries can be directed to the corresponding author/s.

## Ethics Statement

The studies involving human participants were reviewed and approved by Tongde Hospital of Zhejiang Province. The patients/participants provided their written informed consent to participate in this study. Written informed consent was obtained from the individual(s) for the publication of any potentially identifiable images or data included in this article.

## Author Contributions

QH and JS designed this study. QH and YS collected the information, images, and wrote the manuscript. JS reviewed the manuscript. All the authors read and approved the final manuscript.

## Funding

This study was supported by the Science and Technology Planning Project of Zhejiang Province (No. 2017F30045), Science and Technology Planning Project of Traditional Chinese Medicine (No. 2018ZZ004), gastrointestinal surgery of integrated traditional Chinese and Western Medicine (No. 2017-XK-A20).

## Conflict of Interest

The authors declare that the research was conducted in the absence of any commercial or financial relationships that could be construed as a potential conflict of interest.

## Publisher's Note

All claims expressed in this article are solely those of the authors and do not necessarily represent those of their affiliated organizations, or those of the publisher, the editors and the reviewers. Any product that may be evaluated in this article, or claim that may be made by its manufacturer, is not guaranteed or endorsed by the publisher.
